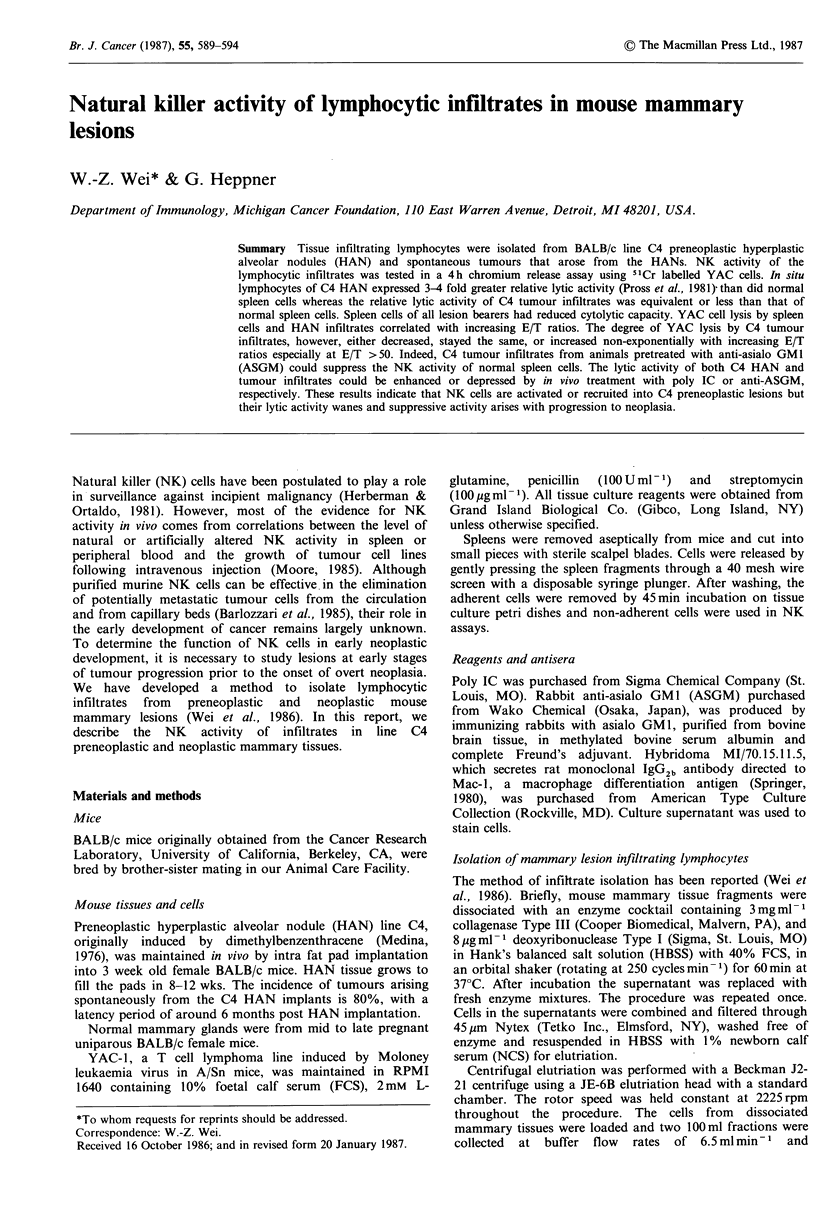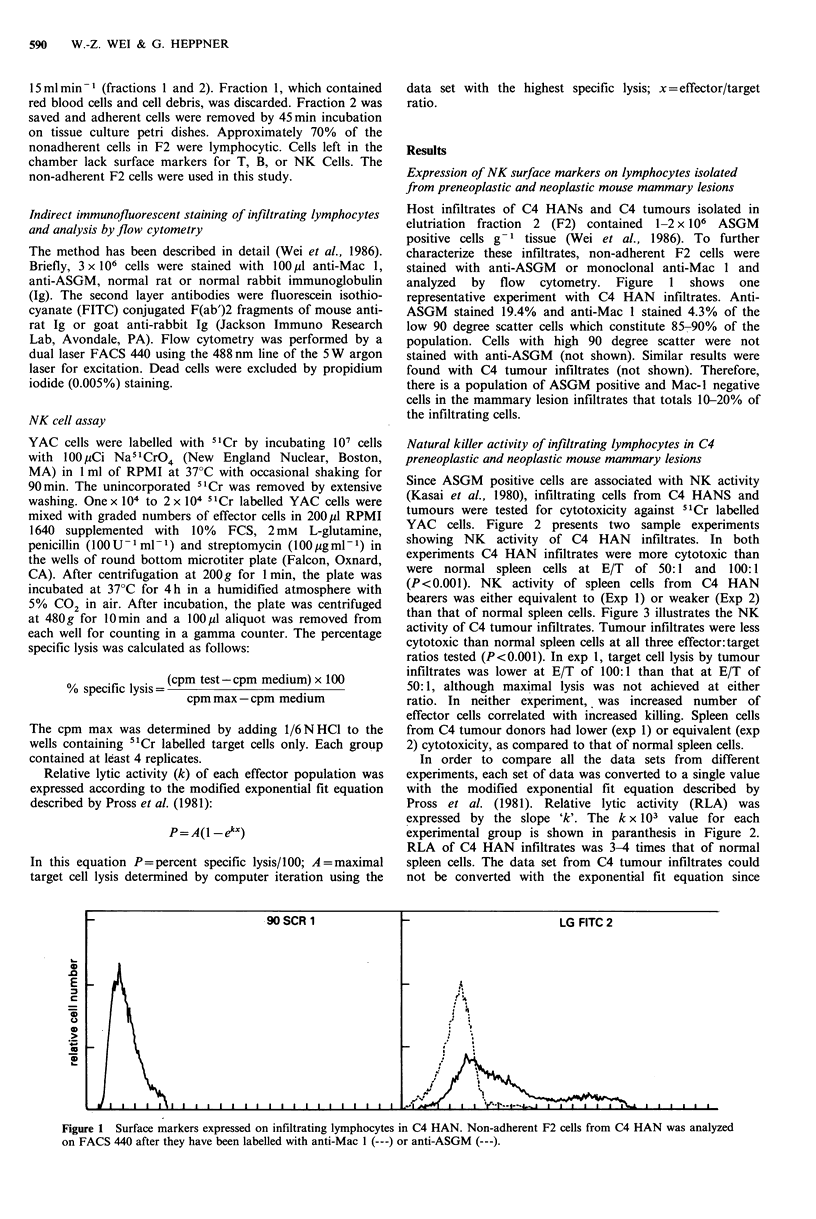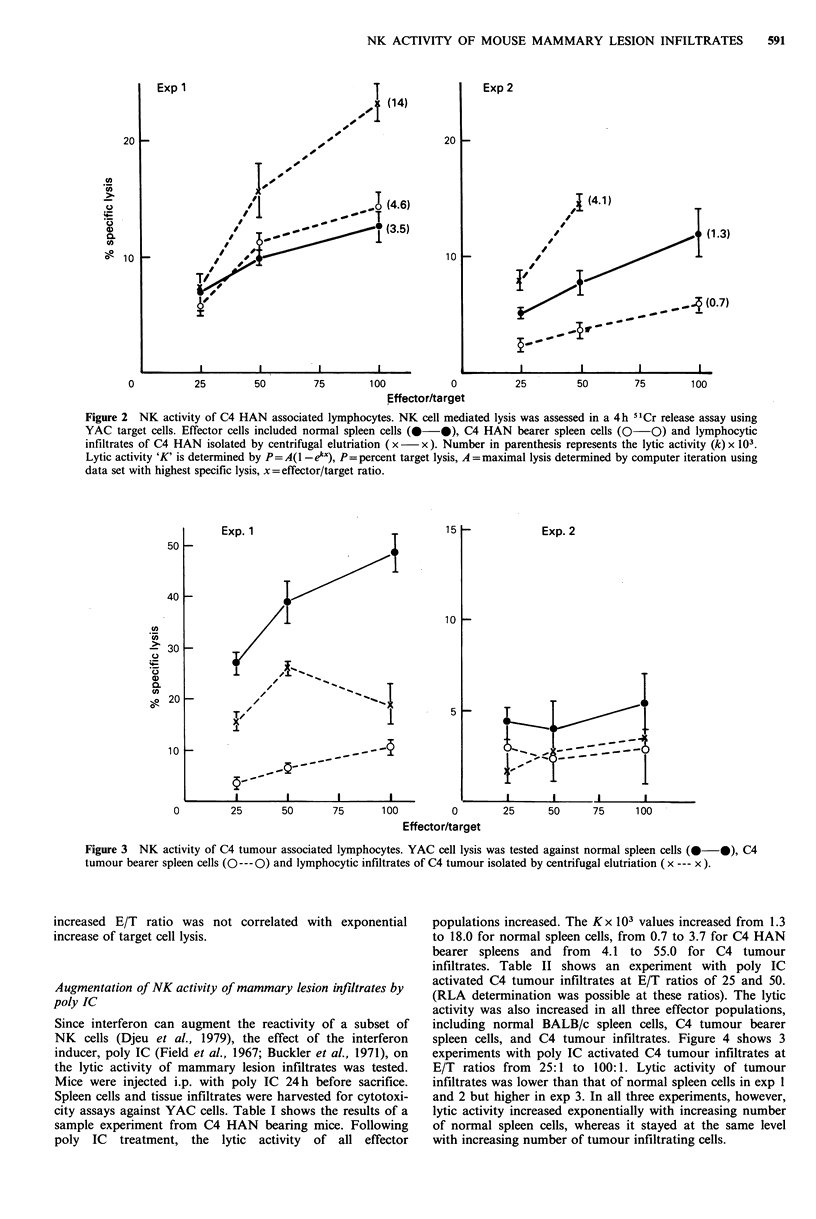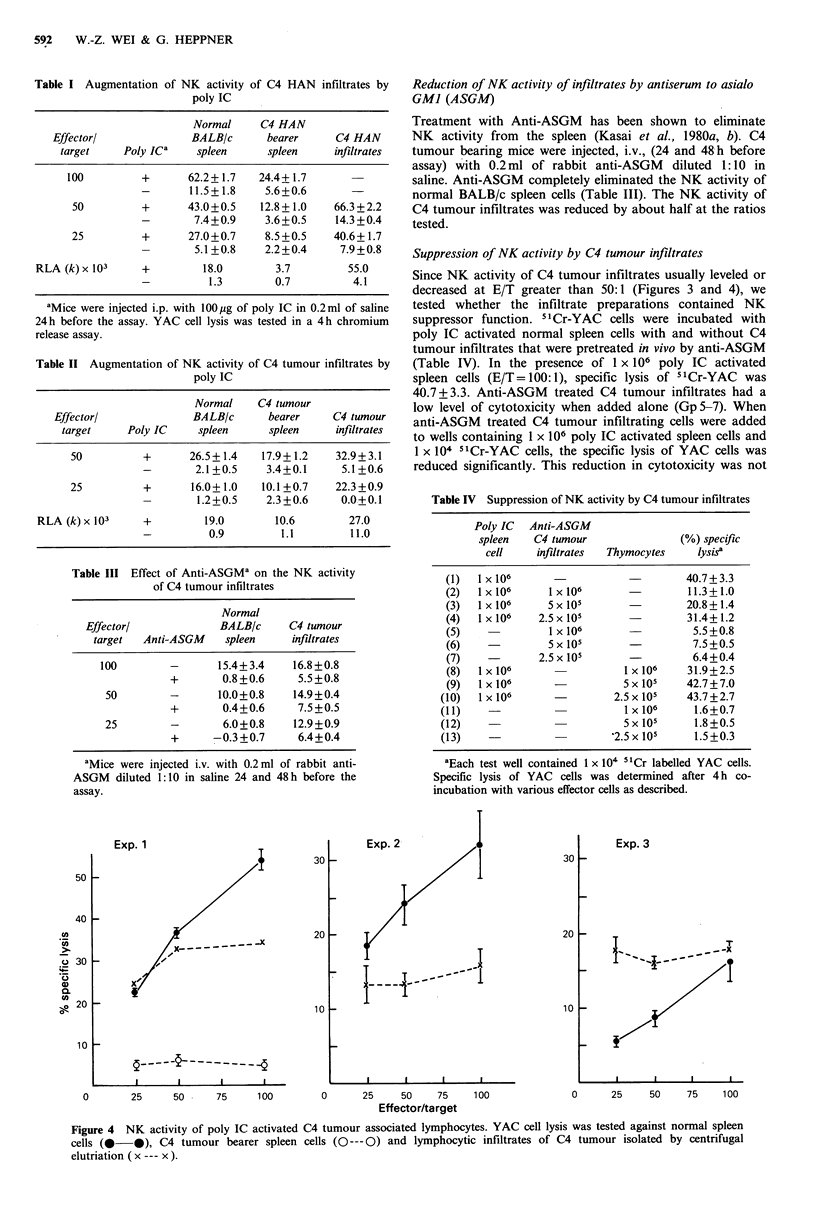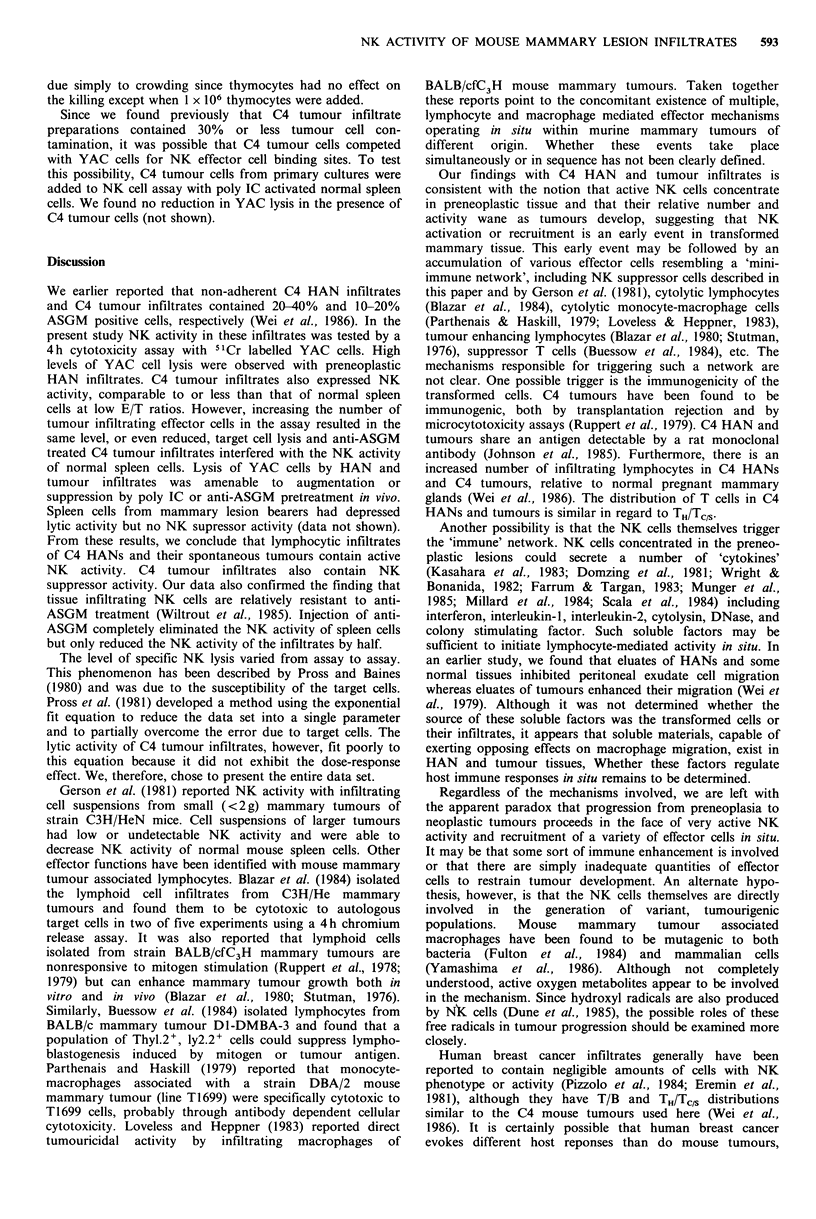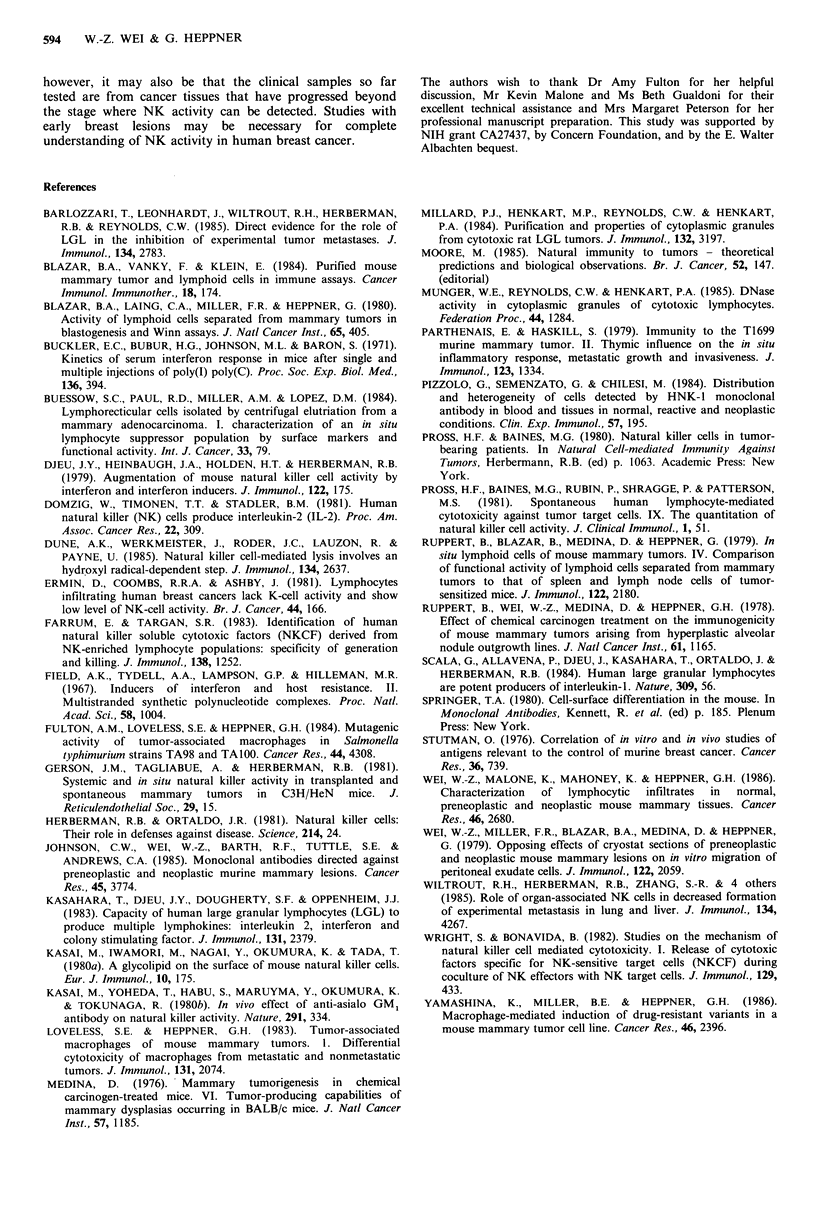# Natural killer activity of lymphocytic infiltrates in mouse mammary lesions.

**DOI:** 10.1038/bjc.1987.120

**Published:** 1987-06

**Authors:** W. Z. Wei, G. Heppner

## Abstract

Tissue infiltrating lymphocytes were isolated from BALB/c line C4 preneoplastic hyperplastic alveolar nodules (HAN) and spontaneous tumours that arose from the HANs. NK activity of the lymphocytic infiltrates was tested in a 4 h chromium release assay using 51Cr labelled YAC cells. In situ lymphocytes of C4 HAN expressed 3-4 fold greater relative lytic activity (Pross et al., 1981) than did normal spleen cells whereas the relative lytic activity of C4 tumour infiltrates was equivalent or less than that of normal spleen cells. Spleen cells of all lesion bearers had reduced cytolytic capacity. YAC cell lysis by spleen cells and HAN infiltrates correlated with increasing E/T ratios. The degree of YAC lysis by C4 tumour infiltrates, however, either decreased, stayed the same, or increased non-exponentially with increasing E/T ratios especially at E/T greater than 50. Indeed, C4 tumour infiltrates from animals pretreated with anti-asialo GM1 (ASGM) could suppress the NK activity of normal spleen cells. The lytic activity of both C4 HAN and tumour infiltrates could be enhanced or depressed by in vivo treatment with poly IC or anti-ASGM, respectively. These results indicate that NK cells are activated or recruited into C4 preneoplastic lesions but their lytic activity wanes and suppressive activity arises with progression to neoplasia.


 © The Macmillan Press Ltd., 1987

### Natural killer activity of lymphocytic infiltrates in mouse mammary lesions

W.-Z. Wei* & G. Heppner

*Department of Immunology, Michigan Cancer Foundation, 110 East Warren Avenue, Detroit, MI 48201, USA.*

**Summary** Tissue infiltrating lymphocytes were isolated from BALB/c line C4 preneoplastic hyperplastic alveolar nodules (HAN) and spontaneous tumours that arose from the HANs. NK activity of the lymphocytic infiltrates was tested in a 4h chromium release assay using $^{51}Cr$ labelled YAC cells. *In situ* lymphocytes of C4 HAN expressed 3–4 fold greater relative lytic activity (Pross *et al.*, 1981) than did normal spleen cells whereas the relative lytic activity of C4 tumour infiltrates was equivalent or less than that of normal spleen cells. Spleen cells of all lesion bearers had reduced cytolytic capacity. YAC cell lysis by spleen cells and HAN infiltrates correlated with increasing E/T ratios. The degree of YAC lysis by C4 tumour infiltrates, however, either decreased, stayed the same, or increased non-exponentially with increasing E/T ratios especially at E/T >50. Indeed, C4 tumour infiltrates from animals pretreated with anti-asialo GM1 (ASGM) could suppress the NK activity of normal spleen cells. The lytic activity of both C4 HAN and tumour infiltrates could be enhanced or depressed by *in vivo* treatment with poly IC or anti-ASGM, respectively. These results indicate that NK cells are activated or recruited into C4 preneoplastic lesions but their lytic activity wanes and suppressive activity arises with progression to neoplasia.

Natural killer (NK) cells have been postulated to play a role in surveillance against incipient malignancy (Herberman & Ortaldo, 1981). However, most of the evidence for NK activity *in vivo* comes from correlations between the level of natural or artificially altered NK activity in spleen or peripheral blood and the growth of tumour cell lines following intravenous injection (Moore, 1985). Although purified murine NK cells can be effective in the elimination of potentially metastatic tumour cells from the circulation and from capillary beds (Barlozzari *et al.*, 1985), their role in the early development of cancer remains largely unknown. To determine the function of NK cells in early neoplastic development, it is necessary to study lesions at early stages of tumour progression prior to the onset of overt neoplasia. We have developed a method to isolate lymphocytic infiltrates from preneoplastic and neoplastic mouse mammary lesions (Wei *et al.*, 1986). In this report, we describe the NK activity of infiltrates in line C4 preneoplastic and neoplastic mammary tissues.

### Materials and methods

### *Mice*

BALB/c mice originally obtained from the Cancer Research Laboratory, University of California, Berkeley, CA, were bred by brother-sister mating in our Animal Care Facility.

### *Mouse tissues and cells*

Preneoplastic hyperplastic alveolar nodule (HAN) line C4, originally induced by dimethylbenzenthracene (Medina, 1976), was maintained *in vivo* by intra fat pad implantation into 3 week old female BALB/c mice. HAN tissue grows to fill the pads in 8–12 wks. The incidence of tumours arising spontaneously from the C4 HAN implants is 80%, with a latency period of around 6 months post HAN implantation.

Normal mammary glands were from mid to late pregnant uniparous BALB/c female mice.

YAC-1, a T cell lymphoma line induced by Moloney leukaemia virus in A/Sn mice, was maintained in RPMI 1640 containing 10% foetal calf serum (FCS), 2 mM L-glutamine, penicillin ($100\,U\,ml^{-1}$) and streptomycin ($100\,\mu g\,ml^{-1}$). All tissue culture reagents were obtained from Grand Island Biological Co. (Gibco, Long Island, NY) unless otherwise specified.

Spleens were removed aseptically from mice and cut into small pieces with sterile scalpel blades. Cells were released by gently pressing the spleen fragments through a 40 mesh wire screen with a disposable syringe plunger. After washing, the adherent cells were removed by 45 min incubation on tissue culture petri dishes and non-adherent cells were used in NK assays.

### *Reagents and antisera*

Poly IC was purchased from Sigma Chemical Company (St. Louis, MO). Rabbit anti-asialo GM1 (ASGM) purchased from Wako Chemical (Osaka, Japan), was produced by immunizing rabbits with asialo GM1, purified from bovine brain tissue, in methylated bovine serum albumin and complete Freund's adjuvant. Hybridoma MI/70.15.11.5, which secretes rat monoclonal $IgG_{2b}$ antibody directed to Mac-1, a macrophage differentiation antigen (Springer, 1980), was purchased from American Type Culture Collection (Rockville, MD). Culture supernatant was used to stain cells.

### *Isolation of mammary lesion infiltrating lymphocytes*

The method of infiltrate isolation has been reported (Wei *et al.*, 1986). Briefly, mouse mammary tissue fragments were dissociated with an enzyme cocktail containing $3\,mg\,ml^{-1}$ collagenase Type III (Cooper Biomedical, Malvern, PA), and $8\,\mu g\,ml^{-1}$ deoxyribonuclease Type I (Sigma, St. Louis, MO) in Hank's balanced salt solution (HBSS) with 40% FCS, in an orbital shaker (rotating at 250 cycles $min^{-1}$) for 60 min at 37°C. After incubation the supernatant was replaced with fresh enzyme mixtures. The procedure was repeated once. Cells in the supernatants were combined and filtered through $45\,\mu m$ Nytex (Tetko Inc., Elmsford, NY), washed free of enzyme and resuspended in HBSS with 1% newborn calf serum (NCS) for elutriation.

Centrifugal elutriation was performed with a Beckman J2-21 centrifuge using a JE-6B elutriation head with a standard chamber. The rotor speed was held constant at 2225 rpm throughout the procedure. The cells from dissociated mammary tissues were loaded and two 100 ml fractions were collected at buffer flow rates of $6.5\,ml\,min^{-1}$ and

*To whom requests for reprints should be addressed.
Correspondence: W.-Z. Wei.
Received 16 October 1986; and in revised form 20 January 1987.

$15\,ml\,min^{-1}$ (fractions 1 and 2). Fraction 1, which contained red blood cells and cell debris, was discarded. Fraction 2 was saved and adherent cells were removed by 45 min incubation on tissue culture petri dishes. Approximately 70% of the nonadherent cells in F2 were lymphocytic. Cells left in the chamber lack surface markers for T, B, or NK Cells. The non-adherent F2 cells were used in this study.

*Indirect immunofluorescent staining of infiltrating lymphocytes and analysis by flow cytometry*

The method has been described in detail (Wei *et al.*, 1986). Briefly, $3 \times 10^6$ cells were stained with $100\,\mu l$ anti-Mac 1, anti-ASGM, normal rat or normal rabbit immunoglobulin (Ig). The second layer antibodies were fluorescein isothiocyanate (FITC) conjugated F(ab')2 fragments of mouse anti-rat Ig or goat anti-rabbit Ig (Jackson Immuno Research Lab, Avondale, PA). Flow cytometry was performed by a dual laser FACS 440 using the 488 nm line of the 5 W argon laser for excitation. Dead cells were excluded by propidium iodide (0.005%) staining.

*NK cell assay*

YAC cells were labelled with $^{51}Cr$ by incubating $10^7$ cells with $100\,\mu Ci$ $Na^{51}CrO_4$ (New England Nuclear, Boston, MA) in 1 ml of RPMI at 37°C with occasional shaking for 90 min. The unincorporated $^{51}Cr$ was removed by extensive washing. One $\times 10^4$ to $2 \times 10^4$ $^{51}Cr$ labelled YAC cells were mixed with graded numbers of effector cells in $200\,\mu l$ RPMI 1640 supplemented with 10% FCS, 2 mM L-glutamine, penicillin ($100\,U^{-1}\,ml^{-1}$) and streptomycin ($100\,\mu g\,ml^{-1}$) in the wells of round bottom microtiter plate (Falcon, Oxnard, CA). After centrifugation at 200 *g* for 1 min, the plate was incubated at 37°C for 4 h in a humidified atmosphere with 5% $CO_2$ in air. After incubation, the plate was centrifuged at 480 *g* for 10 min and a $100\,\mu l$ aliquot was removed from each well for counting in a gamma counter. The percentage specific lysis was calculated as follows:

$$\%\ \text{specific lysis} = \frac{(\text{cpm test} - \text{cpm medium}) \times 100}{\text{cpm max} - \text{cpm medium}}$$

The cpm max was determined by adding 1/6 N HCl to the wells containing $^{51}Cr$ labelled target cells only. Each group contained at least 4 replicates.

Relative lytic activity ($k$) of each effector population was expressed according to the modified exponential fit equation described by Pross *et al.* (1981):

$$P = A(1 - e^{kx})$$

In this equation $P$ = percent specific lysis/100; $A$ = maximal target cell lysis determined by computer iteration using the data set with the highest specific lysis; $x$ = effector/target ratio.

### Results

*Expression of NK surface markers on lymphocytes isolated from preneoplastic and neoplastic mouse mammary lesions*

Host infiltrates of C4 HANs and C4 tumours isolated in elutriation fraction 2 (F2) contained $1–2 \times 10^6$ ASGM positive cells $g^{-1}$ tissue (Wei *et al.*, 1986). To further characterize these infiltrates, non-adherent F2 cells were stained with anti-ASGM or monoclonal anti-Mac 1 and analyzed by flow cytometry. Figure 1 shows one representative experiment with C4 HAN infiltrates. Anti-ASGM stained 19.4% and anti-Mac 1 stained 4.3% of the low 90 degree scatter cells which constitute 85–90% of the population. Cells with high 90 degree scatter were not stained with anti-ASGM (not shown). Similar results were found with C4 tumour infiltrates (not shown). Therefore, there is a population of ASGM positive and Mac-1 negative cells in the mammary lesion infiltrates that totals 10–20% of the infiltrating cells.

*Natural killer activity of infiltrating lymphocytes in C4 preneoplastic and neoplastic mouse mammary lesions*

Since ASGM positive cells are associated with NK activity (Kasai *et al.*, 1980), infiltrating cells from C4 HANS and tumours were tested for cytotoxicity against $^{51}Cr$ labelled YAC cells. Figure 2 presents two sample experiments showing NK activity of C4 HAN infiltrates. In both experiments C4 HAN infiltrates were more cytotoxic than were normal spleen cells at E/T of 50:1 and 100:1 ($P < 0.001$). NK activity of spleen cells from C4 HAN bearers was either equivalent to (Exp 1) or weaker (Exp 2) than that of normal spleen cells. Figure 3 illustrates the NK activity of C4 tumour infiltrates. Tumour infiltrates were less cytotoxic than normal spleen cells at all three effector:target ratios tested ($P < 0.001$). In exp 1, target cell lysis by tumour infiltrates was lower at E/T of 100:1 than that at E/T of 50:1, although maximal lysis was not achieved at either ratio. In neither experiment, was increased number of effector cells correlated with increased killing. Spleen cells from C4 tumour donors had lower (exp 1) or equivalent (exp 2) cytotoxicity, as compared to that of normal spleen cells.

In order to compare all the data sets from different experiments, each set of data was converted to a single value with the modified exponential fit equation described by Pross *et al.* (1981). Relative lytic activity (RLA) was expressed by the slope '$k$'. The $k \times 10^3$ value for each experimental group is shown in paranthesis in Figure 2. RLA of C4 HAN infiltrates was 3–4 times that of normal spleen cells. The data set from C4 tumour infiltrates could not be converted with the exponential fit equation since

**Figure 1** Surface markers expressed on infiltrating lymphocytes in C4 HAN. Non-adherent F2 cells from C4 HAN was analyzed on FACS 440 after they have been labelled with anti-Mac 1 (---) or anti-ASGM (---).

**Figure 2** NK activity of C4 HAN associated lymphocytes. NK cell mediated lysis was assessed in a 4 h $^{51}Cr$ release assay using YAC target cells. Effector cells included normal spleen cells (●——●), C4 HAN bearer spleen cells (○——○) and lymphocytic infiltrates of C4 HAN isolated by centrifugal elutriation (×——×). Number in parenthesis represents the lytic activity $(k) \times 10^3$. Lytic activity '$K$' is determined by $P = A(1 - e^{kx})$, $P$ = percent target lysis, $A$ = maximal lysis determined by computer iteration using data set with highest specific lysis, $x$ = effector/target ratio.

**Figure 3** NK activity of C4 tumour associated lymphocytes. YAC cell lysis was tested against normal spleen cells (●——●), C4 tumour bearer spleen cells (○---○) and lymphocytic infiltrates of C4 tumour isolated by centrifugal elutriation (×---×).

increased E/T ratio was not correlated with exponential increase of target cell lysis.

### *Augmentation of NK activity of mammary lesion infiltrates by poly IC*

Since interferon can augment the reactivity of a subset of NK cells (Djeu *et al.*, 1979), the effect of the interferon inducer, poly IC (Field *et al.*, 1967; Buckler *et al.*, 1971), on the lytic activity of mammary lesion infiltrates was tested. Mice were injected i.p. with poly IC 24 h before sacrifice. Spleen cells and tissue infiltrates were harvested for cytotoxicity assays against YAC cells. Table I shows the results of a sample experiment from C4 HAN bearing mice. Following poly IC treatment, the lytic activity of all effector populations increased. The $K \times 10^3$ values increased from 1.3 to 18.0 for normal spleen cells, from 0.7 to 3.7 for C4 HAN bearer spleens and from 4.1 to 55.0 for C4 tumour infiltrates. Table II shows an experiment with poly IC activated C4 tumour infiltrates at E/T ratios of 25 and 50. (RLA determination was possible at these ratios). The lytic activity was also increased in all three effector populations, including normal BALB/c spleen cells, C4 tumour bearer spleen cells, and C4 tumour infiltrates. Figure 4 shows 3 experiments with poly IC activated C4 tumour infiltrates at E/T ratios from 25:1 to 100:1. Lytic activity of tumour infiltrates was lower than that of normal spleen cells in exp 1 and 2 but higher in exp 3. In all three experiments, however, lytic activity increased exponentially with increasing number of normal spleen cells, whereas it stayed at the same level with increasing number of tumour infiltrating cells.

**Table I** Augmentation of NK activity of C4 HAN infiltrates by poly IC

| *Effector/ target* | *Poly IC*[a] | *Normal BALB/c spleen* | *C4 HAN bearer spleen* | *C4 HAN infiltrates* |
|---|---|---|---|---|
| 100 | + | 62.2±1.7 | 24.4±1.7 | — |
| | – | 11.5±1.8 | 5.6±0.6 | — |
| 50 | + | 43.0±0.5 | 12.8±1.0 | 66.3±2.2 |
| | – | 7.4±0.9 | 3.6±0.5 | 14.3±0.4 |
| 25 | + | 27.0±0.7 | 8.5±0.5 | 40.6±1.7 |
| | – | 5.1±0.8 | 2.2±0.4 | 7.9±0.8 |
| RLA ($k$) × $10^3$ | + | 18.0 | 3.7 | 55.0 |
| | – | 1.3 | 0.7 | 4.1 |

[a]Mice were injected i.p. with 100 $\mu$g of poly IC in 0.2 ml of saline 24 h before the assay. YAC cell lysis was tested in a 4 h chromium release assay.

**Table II** Augmentation of NK activity of C4 tumour infiltrates by poly IC

| *Effector/ target* | *Poly IC* | *Normal BALB/c spleen* | *C4 tumour bearer spleen* | *C4 tumour infiltrates* |
|---|---|---|---|---|
| 50 | + | 26.5±1.4 | 17.9±1.2 | 32.9±3.1 |
| | – | 2.1±0.5 | 3.4±0.1 | 5.1±0.6 |
| 25 | + | 16.0±1.0 | 10.1±0.7 | 22.3±0.9 |
| | – | 1.2±0.5 | 2.3±0.6 | 0.0±0.1 |
| RLA ($k$) × $10^3$ | + | 19.0 | 10.6 | 27.0 |
| | – | 0.9 | 1.1 | 11.0 |

**Table III** Effect of Anti-ASGM[a] on the NK activity of C4 tumour infiltrates

| *Effector/ target* | *Anti-ASGM* | *Normal BALB/c spleen* | *C4 tumour infiltrates* |
|---|---|---|---|
| 100 | – | 15.4±3.4 | 16.8±0.8 |
| | + | 0.8±0.6 | 5.5±0.8 |
| 50 | – | 10.0±0.8 | 14.9±0.4 |
| | + | 0.4±0.6 | 7.5±0.5 |
| 25 | – | 6.0±0.8 | 12.9±0.9 |
| | + | −0.3±0.7 | 6.4±0.4 |

[a]Mice were injected i.v. with 0.2 ml of rabbit anti-ASGM diluted 1:10 in saline 24 and 48 h before the assay.

*Reduction of NK activity of infiltrates by antiserum to asialo GM1 (ASGM)*

Treatment with Anti-ASGM has been shown to eliminate NK activity from the spleen (Kasai *et al.*, 1980*a*, *b*). C4 tumour bearing mice were injected, i.v., (24 and 48 h before assay) with 0.2 ml of rabbit anti-ASGM diluted 1:10 in saline. Anti-ASGM completely eliminated the NK activity of normal BALB/c spleen cells (Table III). The NK activity of C4 tumour infiltrates was reduced by about half at the ratios tested.

*Suppression of NK activity by C4 tumour infiltrates*

Since NK activity of C4 tumour infiltrates usually leveled or decreased at E/T greater than 50:1 (Figures 3 and 4), we tested whether the infiltrate preparations contained NK suppressor function. $^{51}$Cr-YAC cells were incubated with poly IC activated normal spleen cells with and without C4 tumour infiltrates that were pretreated *in vivo* by anti-ASGM (Table IV). In the presence of $1 \times 10^6$ poly IC activated spleen cells (E/T = 100:1), specific lysis of $^{51}$Cr-YAC was 40.7±3.3. Anti-ASGM treated C4 tumour infiltrates had a low level of cytotoxicity when added alone (Gp 5–7). When anti-ASGM treated C4 tumour infiltrating cells were added to wells containing $1 \times 10^6$ poly IC activated spleen cells and $1 \times 10^4$ $^{51}$Cr-YAC cells, the specific lysis of YAC cells was reduced significantly. This reduction in cytotoxicity was not

**Table IV** Suppression of NK activity by C4 tumour infiltrates

| | *Poly IC spleen cell* | *Anti-ASGM C4 tumour infiltrates* | *Thymocytes* | *(%) specific lysis*[a] |
|---|---|---|---|---|
| (1) | $1 \times 10^6$ | — | — | 40.7±3.3 |
| (2) | $1 \times 10^6$ | $1 \times 10^6$ | — | 11.3±1.0 |
| (3) | $1 \times 10^6$ | $5 \times 10^5$ | — | 20.8±1.4 |
| (4) | $1 \times 10^6$ | $2.5 \times 10^5$ | — | 31.4±1.2 |
| (5) | — | $1 \times 10^6$ | — | 5.5±0.8 |
| (6) | — | $5 \times 10^5$ | — | 7.5±0.5 |
| (7) | — | $2.5 \times 10^5$ | — | 6.4±0.4 |
| (8) | $1 \times 10^6$ | — | $1 \times 10^6$ | 31.9±2.5 |
| (9) | $1 \times 10^6$ | — | $5 \times 10^5$ | 42.7±7.0 |
| (10) | $1 \times 10^6$ | — | $2.5 \times 10^5$ | 43.7±2.7 |
| (11) | — | — | $1 \times 10^6$ | 1.6±0.7 |
| (12) | — | — | $5 \times 10^5$ | 1.8±0.5 |
| (13) | — | — | $2.5 \times 10^5$ | 1.5±0.3 |

[a]Each test well contained $1 \times 10^4$ $^{51}$Cr labelled YAC cells. Specific lysis of YAC cells was determined after 4 h co-incubation with various effector cells as described.

**Figure 4** NK activity of poly IC activated C4 tumour associated lymphocytes. YAC cell lysis was tested against normal spleen cells (●——●), C4 tumour bearer spleen cells (O---O) and lymphocytic infiltrates of C4 tumour isolated by centrifugal elutriation (×---×).

due simply to crowding since thymocytes had no effect on the killing except when $1 \times 10^6$ thymocytes were added.

Since we found previously that C4 tumour infiltrate preparations contained 30% or less tumour cell contamination, it was possible that C4 tumour cells competed with YAC cells for NK effector cell binding sites. To test this possibility, C4 tumour cells from primary cultures were added to NK cell assay with poly IC activated normal spleen cells. We found no reduction in YAC lysis in the presence of C4 tumour cells (not shown).

### Discussion

We earlier reported that non-adherent C4 HAN infiltrates and C4 tumour infiltrates contained 20–40% and 10–20% ASGM positive cells, respectively (Wei *et al.*, 1986). In the present study NK activity in these infiltrates was tested by a 4 h cytotoxicity assay with $^{51}Cr$ labelled YAC cells. High levels of YAC cell lysis were observed with preneoplastic HAN infiltrates. C4 tumour infiltrates also expressed NK activity, comparable to or less than that of normal spleen cells at low E/T ratios. However, increasing the number of tumour infiltrating effector cells in the assay resulted in the same level, or even reduced, target cell lysis and anti-ASGM treated C4 tumour infiltrates interfered with the NK activity of normal spleen cells. Lysis of YAC cells by HAN and tumour infiltrates was amenable to augmentation or suppression by poly IC or anti-ASGM pretreatment *in vivo*. Spleen cells from mammary lesion bearers had depressed lytic activity but no NK supressor activity (data not shown). From these results, we conclude that lymphocytic infiltrates of C4 HANs and their spontaneous tumours contain active NK activity. C4 tumour infiltrates also contain NK suppressor activity. Our data also confirmed the finding that tissue infiltrating NK cells are relatively resistant to anti-ASGM treatment (Wiltrout *et al.*, 1985). Injection of anti-ASGM completely eliminated the NK activity of spleen cells but only reduced the NK activity of the infiltrates by half.

The level of specific NK lysis varied from assay to assay. This phenomenon has been described by Pross and Baines (1980) and was due to the susceptibility of the target cells. Pross *et al.* (1981) developed a method using the exponential fit equation to reduce the data set into a single parameter and to partially overcome the error due to target cells. The lytic activity of C4 tumour infiltrates, however, fit poorly to this equation because it did not exhibit the dose-response effect. We, therefore, chose to present the entire data set.

Gerson *et al.* (1981) reported NK activity with infiltrating cell suspensions from small (<2 g) mammary tumours of strain C3H/HeN mice. Cell suspensions of larger tumours had low or undetectable NK activity and were able to decrease NK activity of normal mouse spleen cells. Other effector functions have been identified with mouse mammary tumour associated lymphocytes. Blazar *et al.* (1984) isolated the lymphoid cell infiltrates from C3H/He mammary tumours and found them to be cytotoxic to autologous target cells in two of five experiments using a 4 h chromium release assay. It was also reported that lymphoid cells isolated from strain BALB/cf$C_3$H mammary tumours are nonresponsive to mitogen stimulation (Ruppert *et al.*, 1978; 1979) but can enhance mammary tumour growth both *in vitro* and *in vivo* (Blazar *et al.*, 1980; Stutman, 1976). Similarly, Buessow *et al.* (1984) isolated lymphocytes from BALB/c mammary tumour D1-DMBA-3 and found that a population of Thyl.$2^+$, ly2.$2^+$ cells could suppress lymphoblastogenesis induced by mitogen or tumour antigen. Parthenais and Haskill (1979) reported that monocyte-macrophages associated with a strain DBA/2 mouse mammary tumour (line T1699) were specifically cytotoxic to T1699 cells, probably through antibody dependent cellular cytotoxicity. Loveless and Heppner (1983) reported direct tumouricidal activity by infiltrating macrophages of BALB/cf$C_3$H mouse mammary tumours. Taken together these reports point to the concomitant existence of multiple, lymphocyte and macrophage mediated effector mechanisms operating *in situ* within murine mammary tumours of different origin. Whether these events take place simultaneously or in sequence has not been clearly defined.

Our findings with C4 HAN and tumour infiltrates is consistent with the notion that active NK cells concentrate in preneoplastic tissue and that their relative number and activity wane as tumours develop, suggesting that NK activation or recruitment is an early event in transformed mammary tissue. This early event may be followed by an accumulation of various effector cells resembling a 'mini-immune network', including NK suppressor cells described in this paper and by Gerson *et al.* (1981), cytolytic lymphocytes (Blazar *et al.*, 1984), cytolytic monocyte-macrophage cells (Parthenais & Haskill, 1979; Loveless & Heppner, 1983), tumour enhancing lymphocytes (Blazar *et al.*, 1980; Stutman, 1976), suppressor T cells (Buessow *et al.*, 1984), etc. The mechanisms responsible for triggering such a network are not clear. One possible trigger is the immunogenicity of the transformed cells. C4 tumours have been found to be immunogenic, both by transplantation rejection and by microcytotoxicity assays (Ruppert *et al.*, 1979). C4 HAN and tumours share an antigen detectable by a rat monoclonal antibody (Johnson *et al.*, 1985). Furthermore, there is an increased number of infiltrating lymphocytes in C4 HANs and C4 tumours, relative to normal pregnant mammary glands (Wei *et al.*, 1986). The distribution of T cells in C4 HANs and tumours is similar in regard to $T_H/T_{C/S}$.

Another possibility is that the NK cells themselves trigger the 'immune' network. NK cells concentrated in the preneoplastic lesions could secrete a number of 'cytokines' (Kasahara *et al.*, 1983; Domzing *et al.*, 1981; Wright & Bonanida, 1982; Farrum & Targan, 1983; Munger *et al.*, 1985; Millard *et al.*, 1984; Scala *et al.*, 1984) including interferon, interleukin-1, interleukin-2, cytolysin, DNase, and colony stimulating factor. Such soluble factors may be sufficient to initiate lymphocyte-mediated activity *in situ*. In an earlier study, we found that eluates of HANs and some normal tissues inhibited peritoneal exudate cell migration whereas eluates of tumours enhanced their migration (Wei *et al.*, 1979). Although it was not determined whether the source of these soluble factors was the transformed cells or their infiltrates, it appears that soluble materials, capable of exerting opposing effects on macrophage migration, exist in HAN and tumour tissues, Whether these factors regulate host immune responses *in situ* remains to be determined.

Regardless of the mechanisms involved, we are left with the apparent paradox that progression from preneoplasia to neoplastic tumours proceeds in the face of very active NK activity and recruitment of a variety of effector cells *in situ*. It may be that some sort of immune enhancement is involved or that there are simply inadequate quantities of effector cells to restrain tumour development. An alternate hypothesis, however, is that the NK cells themselves are directly involved in the generation of variant, tumourigenic populations. Mouse mammary tumour associated macrophages have been found to be mutagenic to both bacteria (Fulton *et al.*, 1984) and mammalian cells (Yamashima *et al.*, 1986). Although not completely understood, active oxygen metabolites appear to be involved in the mechanism. Since hydroxyl radicals are also produced by NK cells (Dune *et al.*, 1985), the possible roles of these free radicals in tumour progression should be examined more closely.

Human breast cancer infiltrates generally have been reported to contain negligible amounts of cells with NK phenotype or activity (Pizzolo *et al.*, 1984; Eremin *et al.*, 1981), although they have T/B and $T_H/T_{C/S}$ distributions similar to the C4 mouse tumours used here (Wei *et al.*, 1986). It is certainly possible that human breast cancer evokes different host reponses than do mouse tumours, however, it may also be that the clinical samples so far tested are from cancer tissues that have progressed beyond the stage where NK activity can be detected. Studies with early breast lesions may be necessary for complete understanding of NK activity in human breast cancer.

The authors wish to thank Dr Amy Fulton for her helpful discussion, Mr Kevin Malone and Ms Beth Gualdoni for their excellent technical assistance and Mrs Margaret Peterson for her professional manuscript preparation. This study was supported by NIH grant CA27437, by Concern Foundation, and by the E. Walter Albachten bequest.

### References

BARLOZZARI, T., LEONHARDT, J., WILTROUT, R.H., HERBERMAN, R.B. & REYNOLDS, C.W. (1985). Direct evidence for the role of LGL in the inhibition of experimental tumor metastases. *J. Immunol.*, **134**, 2783.

BLAZAR, B.A., VANKY, F. & KLEIN, E. (1984). Purified mouse mammary tumor and lymphoid cells in immune assays. *Cancer Immunol. Immunother.*, **18**, 174.

BLAZAR, B.A., LAING, C.A., MILLER, F.R. & HEPPNER, G. (1980). Activity of lymphoid cells separated from mammary tumors in blastogenesis and Winn assays. *J. Natl Cancer Inst.*, **65**, 405.

BUCKLER, E.C., BUBUR, H.G., JOHNSON, M.L. & BARON, S. (1971). Kinetics of serum interferon response in mice after single and multiple injections of poly(I) poly(C). *Proc. Soc. Exp. Biol. Med.*, **136**, 394.

BUESSOW, S.C., PAUL, R.D., MILLER, A.M. & LOPEZ, D.M. (1984). Lymphoreticular cells isolated by centrifugal elutriation from a mammary adenocarcinoma. I. characterization of an *in situ* lymphocyte suppressor population by surface markers and functional activity. *Int. J. Cancer*, **33**, 79.

DJEU, J.Y., HEINBAUGH, J.A., HOLDEN, H.T. & HERBERMAN, R.B. (1979). Augmentation of mouse natural killer cell activity by interferon and interferon inducers. *J. Immunol.*, **122**, 175.

DOMZIG, W., TIMONEN, T.T. & STADLER, B.M. (1981). Human natural killer (NK) cells produce interleukin-2 (IL-2). *Proc. Am. Assoc. Cancer Res.*, **22**, 309.

DUNE, A.K., WERKMEISTER, J., RODER, J.C., LAUZON, R. & PAYNE, U. (1985). Natural killer cell-mediated lysis involves an hydroxyl radical-dependent step. *J. Immunol.*, **134**, 2637.

ERMIN, D., COOMBS, R.R.A. & ASHBY, J. (1981). Lymphocytes infiltrating human breast cancers lack K-cell activity and show low level of NK-cell activity. *Br. J. Cancer*, **44**, 166.

FARRUM, E. & TARGAN, S.R. (1983). Identification of human natural killer soluble cytotoxic factors (NKCF) derived from NK-enriched lymphocyte populations: specificity of generation and killing. *J. Immunol.*, **138**, 1252.

FIELD, A.K., TYDELL, A.A., LAMPSON, G.P. & HILLEMAN, M.R. (1967). Inducers of interferon and host resistance. II. Multistranded synthetic polynucleotide complexes. *Proc. Natl. Acad. Sci.*, **58**, 1004.

FULTON, A.M., LOVELESS, S.E. & HEPPNER, G.H. (1984). Mutagenic activity of tumor-associated macrophages in *Salmonella typhimurium* strains TA98 and TA100. *Cancer Res.*, **44**, 4308.

GERSON, J.M., TAGLIABUE, A. & HERBERMAN, R.B. (1981). Systemic and *in situ* natural killer activity in transplanted and spontaneous mammary tumors in C3H/HeN mice. *J. Reticulendothelial Soc.*, **29**, 15.

HERBERMAN, R.B. & ORTALDO, J.R. (1981). Natural killer cells: Their role in defenses against disease. *Science*, **214**, 24.

JOHNSON, C.W., WEI, W.-Z., BARTH, R.F., TUTTLE, S.E. & ANDREWS, C.A. (1985). Monoclonal antibodies directed against preneoplastic and neoplastic murine mammary lesions. *Cancer Res.*, **45**, 3774.

KASAHARA, T., DJEU, J.Y., DOUGHERTY, S.F. & OPPENHEIM, J.J. (1983). Capacity of human large granular lymphocytes (LGL) to produce multiple lymphokines: interleukin 2, interferon and colony stimulating factor. *J. Immunol.*, **131**, 2379.

KASAI, M., IWAMORI, M., NAGAI, Y., OKUMURA, K. & TADA, T. (1980*a*). A glycolipid on the surface of mouse natural killer cells. *Eur. J. Immunol.*, **10**, 175.

KASAI, M., YOHEDA, T., HABU, S., MARUYMA, Y., OKUMURA, K. & TOKUNAGA, R. (1980*b*). *In vivo* effect of anti-asialo $GM_1$ antibody on natural killer activity. *Nature*, **291**, 334.

LOVELESS, S.E. & HEPPNER, G.H. (1983). Tumor-associated macrophages of mouse mammary tumors. 1. Differential cytotoxicity of macrophages from metastatic and nonmetastatic tumors. *J. Immunol.*, **131**, 2074.

MEDINA, D. (1976). Mammary tumorigenesis in chemical carcinogen-treated mice. VI. Tumor-producing capabilities of mammary dysplasias occurring in BALB/c mice. *J. Natl Cancer Inst.*, **57**, 1185.

MILLARD, P.J., HENKART, M.P., REYNOLDS, C.W. & HENKART, P.A. (1984). Purification and properties of cytoplasmic granules from cytotoxic rat LGL tumors. *J. Immunol.*, **132**, 3197.

MOORE, M. (1985). Natural immunity to tumors – theoretical predictions and biological observations. *Br. J. Cancer*, **52**, 147. (editorial)

MUNGER, W.E., REYNOLDS, C.W. & HENKART, P.A. (1985). DNase activity in cytoplasmic granules of cytotoxic lymphocytes. *Federation Proc.*, **44**, 1284.

PARTHENAIS, E. & HASKILL, S. (1979). Immunity to the T1699 murine mammary tumor. II. Thymic influence on the *in situ* inflammatory response, metastatic growth and invasiveness. *J. Immunol.*, **123**, 1334.

PIZZOLO, G., SEMENZATO, G. & CHILESI, M. (1984). Distribution and heterogeneity of cells detected by HNK-1 monoclonal antibody in blood and tissues in normal, reactive and neoplastic conditions. *Clin. Exp. Immunol.*, **57**, 195.

PROSS, H.F. & BAINES, M.G. (1980). Natural killer cells in tumor-bearing patients. In *Natural Cell-mediated Immunity Against Tumors*, Herbermann, R.B. (ed) p. 1063. Academic Press: New York.

PROSS, H.F., BAINES, M.G., RUBIN, P., SHRAGGE, P. & PATTERSON, M.S. (1981). Spontaneous human lymphocyte-mediated cytotoxicity against tumor target cells. IX. The quantitation of natural killer cell activity. *J. Clinical Immunol.*, **1**, 51.

RUPPERT, B., BLAZAR, B., MEDINA, D. & HEPPNER, G. (1979). *In situ* lymphoid cells of mouse mammary tumors. IV. Comparison of functional activity of lymphoid cells separated from mammary tumors to that of spleen and lymph node cells of tumor-sensitized mice. *J. Immunol.*, **122**, 2180.

RUPPERT, B., WEI, W.-Z., MEDINA, D. & HEPPNER, G.H. (1978). Effect of chemical carcinogen treatment on the immunogenicity of mouse mammary tumors arising from hyperplastic alveolar nodule outgrowth lines. *J. Natl Cancer Inst.*, **61**, 1165.

SCALA, G., ALLAVENA, P., DJEU, J., KASAHARA, T., ORTALDO, J. & HERBERMAN, R.B. (1984). Human large granular lymphocytes are potent producers of interleukin-1. *Nature*, **309**, 56.

SPRINGER, T.A. (1980). Cell-surface differentiation in the mouse. In *Monoclonal Antibodies*, Kennett, R. *et al.* (ed) p. 185. Plenum Press: New York.

STUTMAN, O. (1976). Correlation of *in vitro* and *in vivo* studies of antigens relevant to the control of murine breast cancer. *Cancer Res.*, **36**, 739.

WEI, W.-Z., MALONE, K., MAHONEY, K. & HEPPNER, G.H. (1986). Characterization of lymphocytic infiltrates in normal, preneoplastic and neoplastic mouse mammary tissues. *Cancer Res.*, **46**, 2680.

WEI, W.-Z., MILLER, F.R., BLAZAR, B.A., MEDINA, D. & HEPPNER, G. (1979). Opposing effects of cryostat sections of preneoplastic and neoplastic mouse mammary lesions on *in vitro* migration of peritoneal exudate cells. *J. Immunol.*, **122**, 2059.

WILTROUT, R.H., HERBERMAN, R.B., ZHANG, S.-R. & 4 others (1985). Role of organ-associated NK cells in decreased formation of experimental metastasis in lung and liver. *J. Immunol.*, **134**, 4267.

WRIGHT, S. & BONAVIDA, B. (1982). Studies on the mechanism of natural killer cell mediated cytotoxicity. I. Release of cytotoxic factors specific for NK-sensitive target cells (NKCF) during coculture of NK effectors with NK target cells. *J. Immunol.*, **129**, 433.

YAMASHINA, K., MILLER, B.E. & HEPPNER, G.H. (1986). Macrophage-mediated induction of drug-resistant variants in a mouse mammary tumor cell line. *Cancer Res.*, **46**, 2396.